# The Congress

**Published:** 1887-10

**Authors:** 


### THE CONGRESS.

The Ninth International Medical Congress is now a thing of the past. In attempting anything like a review of the work done by it, one is met by many embarrassments. The dissensions connected with its organization handicapped it from the start. There is no denying the fact that the list of those who became disaffected contained a large proportion of the names which, in medicine, were the best known in Europe and America. It was worse than folly to assert that they would not be missed, or that a Congress without their aid could be as successful as if their coöperation were secured. The revolutionary proceedings at New Orleans, in 1885, put the management in the hands of the West and South, but it forfeited the support of the East, and without that section a really great representation, either in point of numbers or influence, could not be expected from across the seas. Ah! What might not the Ninth Medical Congress have done had not some of the best men in medicine been unnecessarily alienated?

Yet, despite these drawbacks, so unnecessary and so damaging, the meeting was a comparative success. Those who became responsible put forth their most earnest efforts, and worked with a zeal and determination that could not but bear fruit. The number in attendance was not overwhelmingly large, yet it was something more than respectable. There were few names of commanding eminence upon the list, but there were very many who occupy a high position in medicine. The Congress was not composed of acknowledged scientists, but it was mainly made up of intellectual and intelligent men. There were no papers read which will challenge the attention of the world, but there were many that were instructive and thoughtful. Neither in point of numbers in attendance, nor scientific value in the results attained, was it equal to the London meeting of 1881, but it excelled most of the other meetings which have been held. There was practically no restriction upon membership, and any one who chose to invest the ten dollars was permitted to register, yet fewer acknowledged quacks and irregulars availed themselves of the privilege than might have been expected. The total registration was 2,755. That of the London meeting, the largest ever held, was 3,182.

But if there was any difference of opinion concerning the literary and scientific value of the meeting, no one questions its social success. The good feeling and cordiality was very pronounced. The receptions and conversaziones, the garden parties and dinners, were numerous and thoroughly enjoyed. Then there was the excursion to Mount Vernon, upon Government steamers, and the grand excursion tendered to foreign members, from Washington to Niagara and return, all expenses being paid. This was equal to an excursion from London to the Trosachs, had such been given in 1881. The only social regret was that no one had time enough to make the most of the many enjoyable occasions.

The Dental and Oral Section was the largest in the Congress; its sessions were more largely attended, it did more work, and raised and expended more money than any other of the eighteen sections. Nearly 500 persons were registered in this Section alone. At its opening session 420 persons were present by actual count, and the daily average of attendance was 180. Some of the other sections were, at times, obliged to adjourn for want of a sufficient number to make up a meeting. Eleven regular sessions of the dental section were held, and there was never any lack of work to do. Besides all this, the Section held daily clinics, at which from twenty to fifty operators demonstrated.

The general summing up of the work accomplished by the Section shows something to be regretted, and much for which it is to be congratulated. A very few of the worst of the advertising dentists of the country gained admission; but that was not the fault of the Section, for its officers begged permission to station with the general secretary one of its own secretaries, that he might scrutinize those dentists who applied, and thus avoid the intrusion of men who would bring discredit upon us, but this very proper petition was denied. Apparently, the general management wanted as many dollars as were to be obtained, and was not over particular as to who tendered them.

The work of the Section Committees was generally well done. The finances were admirably managed, and that committee deserves the highest commendation. The Reception Committee did little until the second day of the meeting, but after that it attended carefully to its duties. The Clinic Committees accomplished their task admirably, and the chief interest of the meeting was in the work done at the Franklin School building. The rooms for meetings were exceedingly well selected, and nothing could have been better adapted to the purpose of the mechanical and operative displays than the school building engaged, from which all the seats and desks in the different rooms of the three stories had been removed. There was, however, at times, a scarcity of patients for the operative department. The Committee of Supervision proved to be an embarrassment and an encumbrance, and was at once abolished by general consent.

The entertainments consisted of banquets, receptions. excursions and conversaziones. Section XVII gave excursions, receptions, dinners and lunches to its foreign guests, aside from those of the general Congress, and altogether, those from foreign countries who did us the honor to visit us, had no occasion to complain of neglect.

It was, perhaps, to be expected that those who had some ulterior object in writing a paper should be the first to offer themselves, and accordingly there was some mercantile object very plainly perceptible in more than one of the essays. All the papers were said to have been submitted to the criticism of a committee empowered to accept, reject, condense or edit them. We do not know who, if any one, served upon this committee, but we must say that the results deserved the severest reprobation. The veriest trash was admitted, and papers whose advertising objects were scarcely veiled. Precious time was wasted over masses of verbiage in which the few ideas were as two grains of wheat hid in two bushels of chaff. Excellent and scholarly essays were either excluded altogether, read by title, or squeezed into the last few moments of the meeting, while long and tiresome disquisitions, of which only an abstract should have been admitted, were read and discussed at full length.

The meeting needed a firm, strong, resolute governing hand, that should sternly exclude irrelevant matter and hold it strictly and unwaveringly to the consideration of the point before it. But despite these drawbacks, some of which were unavoidable, there is no doubt that the influence of the meeting will be powerful for good. The general tone was excellent, and we do not believe that one person attended it who did not come away with an increased respect for his profession and a firm determination to labor for its advancement. Perfection was not to be expected, and while an honest sense of duty compels criticism upon some points, a sincere exultation in what of good was accomplished will always enable every member to recollect with pride that he too was a member of the Ninth International Medical Congress. But the results obtained were not secured without great labor, and Dr. Taft and the Secretaries, with many of the Vice-Presidents and members of the Council, whom to name would be invidious, deserve the thanks of all dentists for their unwearied efforts to promote the interests of the Congress and the Section. It is not probable that in America the intimate relation existing between dentistry and medicine will ever again be disputed, nor will regular graduates in dentistry ever again be excluded from any important medical meeting, and this result will repay all the toil and expense attendant upon the late memorable meeting.

